# Mechanical Behavior of 3D-Printed Thickness Gradient Honeycomb Structures

**DOI:** 10.3390/ma17122928

**Published:** 2024-06-14

**Authors:** Dongxia Yang, Lihua Guo, Changsheng Fan

**Affiliations:** 1 Key Laboratory of Heilongjiang Underground Engineering Technology, Harbin University, Harbin 150086, China; ydx1976@hrbu.edu.cn (D.Y.); guolihua@hrbu.edu.cn (L.G.); 2 Laboratory of Bio-Based Material Science &Technology of Ministry of Education, College of Computer and Control Engineering, Northeast Forestry University, Harbin 150040, China

**Keywords:** thickness gradient, cellular structure, wall thickness of cell, compressive properties

## Abstract

In order to obtain a lightweight, high-strength, and customizable cellular structure to meet the needs of modern production and life, the mechanical properties of four thickness gradient honeycomb structures were studied. In this paper, four types of honeycomb structure specimens with the same porosity and different Poisson’s ratios were designed and manufactured by using SLA 3D-printing technology, including the honeycomb, square honeycomb, quasi-square honeycomb, and re-entrant honeycomb structures. Based on the plane compression mechanical properties and failure mode analysis of these specimens, the thickness gradient is applied to the honeycomb structure, and four structural forms of the thickness gradient honeycomb structure are formed. The experimental results show that the thickness gradient honeycomb structure exhibits better mechanical properties than the honeycomb structure with a uniform cellular wall thickness. In the studied thickness gradient honeycomb structure, the mechanical properties of the whole structure can be significantly improved by increasing the thickness of cell walls at the upper and lower ends of the structure. The wall thickness, arrangement order, shape, and Poisson’s ratio of the cell all have a significant impact on the mechanical properties of the specimens. These results provide an effective basis for the design and application of cellular structures in the future.

## 1. Introduction

At present, most of the honeycomb structures studied by researchers have the characteristic of a periodic distribution. Honeycomb structures have excellent characteristics such as a low relative density, high overall stiffness and strength, and strong energy absorption capacity [1]. Nowadays, honeycomb structures have been applied in multiple fields of production and daily life, such as the aerospace field [2], ships [3], architecture [4], automobiles, and packaging [5]. Honeycomb structures not only have a high strength and stiffness, but also excellent energy absorption characteristics and impact resistance [6]. If the surface of the honeycomb structure is made smooth and flat, and the internal structure is made in various forms, it will also have good vibration reduction characteristics and thermal insulation and sound absorption performance [7]. The use of honeycomb structures in temporary buildings and automotive components [8,9] can enhance the safety of residents and pedestrians. Honeycomb structures can absorb energy, resist impacts, and protect personal and property safety in the event of accidents.

There are many configurations of honeycomb structural materials, and the most studied ones are foam [10], hexagon [11], quadrilateral [12], corrugated [13], inner hexagon [14], mixed form [15], lattice structure [16], etc. The mechanical properties of honeycomb structures are influenced by various factors such as the raw materials, diversity of the structural topology, and arrangement of the cells. Luo et al. used aluminum materials to study the local impact resistance of honeycomb structures with different Poisson’s ratios, and found that negative-Poisson’s-ratio honeycomb structures have a strong impact bearing capacity, while zero-Poisson’s-ratio honeycomb structures have strong damping and energy absorption capabilities [17]. Xiaobo Gong et al. used finite simulation methods to study the cellular topology of the convex, re-entrant, and semi-re-entrant structures. These structures have typical positive, negative, and zero Poisson’s ratios. The influence of the Poisson’s ratio on the impact resistance of honeycomb structures was explored, and the study showed that positive-Poisson’s-ratio honeycomb structures have a better local impact protection ability [18]. Liu et al. proposed a zero-Poisson’s-ratio honeycomb structure consisting of concave hexagonal structures and square connecting walls, which exhibits axial symmetry and central symmetry. Polyurethane composite material specimens were manufactured using fused deposition modeling (FDM) 3D-printing technology, and their mechanical properties were analyzed. The research results indicate that the mechanical properties of honeycomb structures can be adjusted by changing the geometric parameters [19]. Lira et al. applied ABS materials to study the transverse shear stiffness of hexagonal and thickness gradient hexagonal honeycomb structures, as well as tensile expansion and thickness gradient tensile expansion honeycomb structures. The research results indicate that the changing thickness gradient geometric structure can achieve the optimal stiffness-to-weight ratio of the structure, and can also improve the thermal conductivity and dielectric performance of the structure [20].

The Poisson’s ratio is one of the most important physical quantities with which to characterize the elastic deformation of materials. Different Poisson’s ratios can cause changes in the shear modulus, compressive performance, fracture toughness, energy absorption capacity, and other aspects of structural materials. Through research, it has been found that the Poisson’s ratio of the honeycomb structural materials is determined by the geometric shape and deformation mechanism of the internal material structure [21,22]. The cellular configuration of the honeycomb structure affects the Poisson’s ratio of the honeycomb structure materials [23]. The Poisson’s ratio of the honeycomb structural materials can take positive, zero, and negative values. Materials with different Poisson’s ratios exhibit different behaviors in terms of compressive performance, energy absorption characteristics, elastic modulus, and fracture toughness.

At present, researchers mostly use fused deposition modeling (FDM) and stereo lithography appearance (SLA) methods for the preparation of honeycomb structures. FDM and SLA both belong to the category of additive manufacturing technology, which constructs objects by adding materials layer by layer, and both are usually printed in STL format. Both printing methods will affect the mechanical properties, dimensional accuracy, and overall quality of printed specimens from three aspects: material characteristics, equipment errors, and process parameters. The printing materials for FDM are usually thermoplastic materials, mostly in the form of fine filaments, which are printed by high-temperature melting. The printing material for SLA is liquid resin, which solidifies the resin into a specimen through a polymerization reaction triggered by light. The structure of FDM’s printing equipment mainly includes parts such as the nozzle, printing platform, and motion system. The structure of the nozzle, the control of the ambient temperature inside the printer, and the temperature of the printing platform all affect the fluidity and crystallization process of the printing material, thereby affecting the mechanical properties, appearance, and dimensional accuracy of the specimen. The structure of SLA’s printing equipment mainly includes the resin tank, laser system, printing platform, and motion system. The diameter of the laser spot, scanning speed, scanning spacing, and post-processing technology of the specimen can all affect the mechanical properties, dimensional accuracy, and overall quality of the printed specimens. The FDM method mainly affects the performance of printed specimens in terms of process parameters, including the layer height, printing temperature, printing speed, layer resolution, and direction of specimen construction [24,25]. The printing temperature includes the nozzle temperature, printing environment temperature, and printing platform temperature, and temperature has a significant impact on the fuse speed, melt fluidity, and layer thickness [26]. These result in the FDM-printed specimens having more obvious layering, and the surface of the specimens is relatively rough. The SLA method mainly affects the performance of printed specimens in terms of process parameters, which is manifested in the fact that the laser spot diameter during the liquid curing process will cause the contour of the specimen to increase by one spot radius, resulting in the positive deviation of the specimen. The reasonable selection of the scanning speed and scanning spacing parameters will not affect the surface quality of the specimen, but will reduce the shrinkage and warping deformation of the specimen. Usually, SLA printing is superior to FDM printing in terms of layer thickness accuracy and material filling density. FDM is suitable for producing parts with rough structures, while SLA is suitable for parts with a high precision and smooth surfaces.

The above research indicates that researchers have conducted detailed studies on single-Poisson’s-ratio honeycomb structures, but there are also some issues. Firstly, the porosity of the studied structure varies; secondly, most researchers use FDM 3D-printing technology; and, furthermore, there is relatively little research on the performance of gradient thickness honeycomb structures. Therefore, this article investigates gradient thickness honeycomb structures with the same porosity and different Poisson’s ratios. The thickness variation of the cellular wall of honeycomb is small, which requires a high printing accuracy. This article uses photosensitive resin DSM8000 as the printing material and applies SLA 3D-printing technology. We print gradient thickness honeycomb structure specimens with different Poisson’s ratios. By analyzing the mechanical properties of these specimens, the physical properties of the structural materials can be artificially controlled, thereby obtaining new functional materials.

The summary of this study is as follows: Firstly, four honeycomb structures with the same porosity were designed. Secondly, using SLA 3D-printing technology, honeycomb, square honeycomb, quasi-square honeycomb and re-entrant honeycomb structures are prepared using photosensitive resin materials. Thirdly, we conduct quasi-static compression experiments on the honeycomb structure specimens, analyze their mechanical properties, and explore the influence of gradient thickness on the performance of honeycomb structures with different Poisson’s ratios.

## 2. Materials and Methods

### 2.1. Design and Modelling of the Model

**Figure 1:**
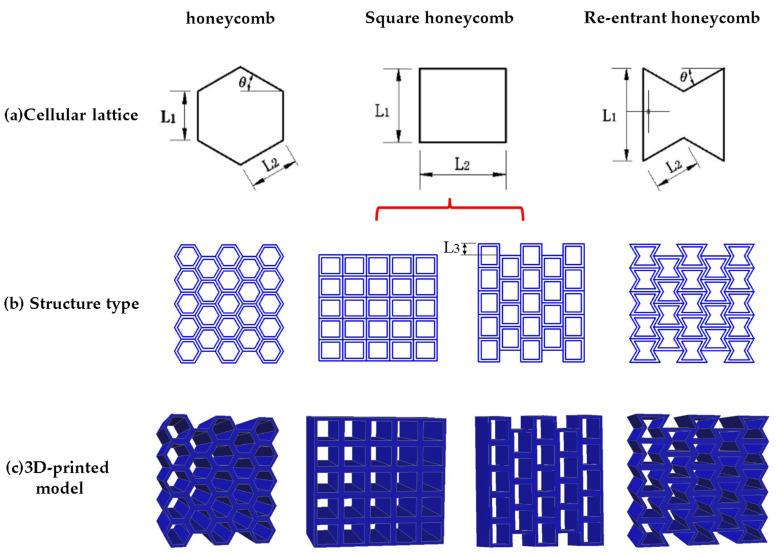
Schematics of specimens, consisting of 4 × 4 units with honeycomb, and square and re-entrant honeycomb. Note: All specimens have a length of 50 mm.

In this paper, the AutoCAD2019 software (version number P.46.0.0) is used to model the honeycomb structure, and the honeycomb, square, and re-entrant hexagon are selected as the models. The three structures have positive Poisson’s ratio, zero Poisson’s ratio, and negative Poisson’s ratio, respectively. The cellular contour areas of the three structures are equal; that is, the three cellular structures have the same porosity. The square with zero Poisson’s ratio can form regular square honeycomb structure and quasi-square honeycomb structure according to the different arrangement. Red line indication in Figure 1a. As shown in Figure 1, L_3_ is equal to 1/2 of L_1_. The structural dimensions of the designed cellular configurations are presented in Table 1.

The cellular configuration and 3D-printed model of honeycomb structures studied in this paper are shown in Figure 1.

### 2.2. Specimen Preparation

**Figure 2:**
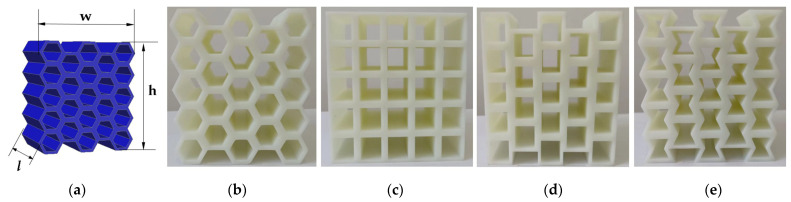
Printed specimens: (**a**) dimensional diagram; (**b**) hexagonal honeycomb; (**c**) square honeycomb; (**d**) quasi-square honeycomb; and (**e**) re-entrant hexagonal honeycomb.

The designed specimens’ materials were made of photosensitive resin (DSM8000) and manufactured by 3D printer (iSLA660, Shenyang, China). The printer has 100% filler and is a solid pillar. The specimens were printed in a manner that is perpendicular to the construction board, so as to avoid the impact on the quality of the specimens due to the lack of support materials along the printing direction. The printed specimens’ model is created in Autocad2019 (version number P.46.0.0) and output in STL rapid prototyping format to zero68y software (version number 6.0.0) for printing. The printed specimens are shown in Figure 2. The print size of the specimens is shown in Table 2. The printing parameters of the specimens are presented in Table 3.

The principle of SLA printing is to apply a laser beam to slice the model, scanning each layer of model slice one by one from point to line, and then to surface. Then, the laser beam irradiates the photosensitive resin to quickly solidify and print the model. This process uses low-power lasers and photopolymer materials to convert photosensitive liquids into 3D solids layer by layer. Therefore, the 3D-printed specimens have anisotropy. In order to avoid the influence of layer direction on the mechanical properties of the specimens, the printed specimens in this study were printed in the “*l*” direction shown in Figure 2.

Considering the influence of the curing saturation of the material on the quality of the specimens, the prepared specimens were kept at room temperature for 7 days to ensure the reliability of the experimental results. Five specimens of each type were prepared.

### 2.3. Raw Material Mechanical Properties

Photosensitive resin, also known as UV-curable resin, is composed of monomers, oligomers, crosslinking agents, and photoinitiators. Monomers are usually low-molecular-weight polymers containing unsaturated double bonds or active groups, which can be polymerized through chemical reactions under the action of photoinitiators. Oligomer is the main component of photosensitive resin. Crosslinking agent is a low-molecular-weight compound containing active functional groups that can react with oligomers or monomers to increase the crosslinking density of polymers. Photoinitiator is a key component in photosensitive resins, which can absorb ultraviolet light, generate free radicals or cations, and initiate monomer polymerization reactions. When the photosensitive resin is exposed to light, the photosensitive substance is activated, triggering a chain-like chemical reaction of the monomer, and the oligomer molecules are connected to each other through chain growth reaction. Thus, a large number of small-molecule monomers or oligomers are linked together to form highly crosslinked polymers. At the same time, the crosslinking agents react with the oligomers or monomers to increase the crosslinking density of the polymer, thereby improving the hardness, strength, and chemical resistance of the photosensitive resin.

In this experiment, the DSM8000 photosensitive resin used was a low-viscosity photosensitive resin material developed by DSMSomos Company, Heerlen, The Netherlands. DSM8000 belongs to the DSM-AGI epoxy acrylic ester series, characterized by low viscosity and opaque white color. Using the iSLA660 3D printer for printing, the liquid photosensitive resin can undergo rapid photopolymerization reaction under the irradiation of a UV laser beam with a wavelength of λ of 354.7 nm, resulting in a sharp increase in molecular weight and a transformation of the material from liquid to solid.

The compression, bending, and tensile properties of the photosensitive resin DSM8000 are measured according to ISO 604-2002 [27], ISO 178-2010 [28], and ASTM D638-2014 standards [29]. A quasi-static compression test was conducted at room temperature using a universal mechanical testing machine at a displacement rate of 0.5 mm/min. The mechanical properties of photosensitive resin DSM8000 are presented in Table 4 [30].

## 3. Experiments

According to the ASTM C365-16 standard [31], uniaxial quasi-static compression tests were carried out on the specimens at room temperature using a general mechanical testing machine (Model WDW-50, Changchun Kexin Testing Instrument Co., Ltd., Changchun, China) at a displacement rate of 1 mm/min. According to the peak value of the displacement–force curve, the experimental value of the structural bearing capacity is obtained. The experimental value of the equivalent compressive elastic modulus is calculated by using the linear interval of the displacement–force curve.

## 4. Analytical Structure

### 4.1. Theoretical Analysis

**Figure 3:**
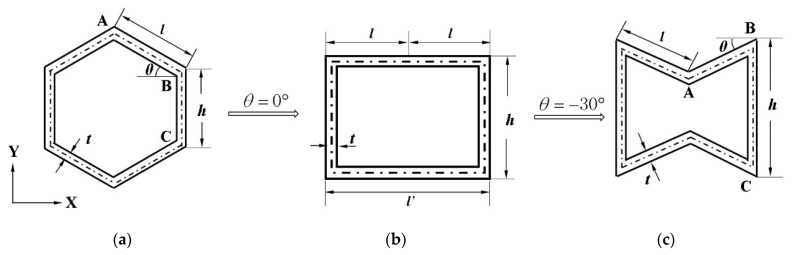
Schematic diagram of hexagonal honeycomb cell evolution process. (**a**) hexagonal honeycomb cell; (**b**) square honeycomb cell; and (**c**) re-entrant hexagonal honeycomb cell.

A detailed study of the cells of the honeycomb structure shows that, when *θ* = 0°, the hexagonal honeycomb cell can change into a square, and, when *θ* is negative, it can change into a re-entrant honeycomb, as shown in Figure 3. Figure 3 shows the effect of angle *θ* on the three cell structures. The length of the cellular wall is set to a special value. The geometric parameters of the cellular structure studied in this paper are shown in Table 1.

Gibson et al. analyzed the static mechanical properties of hexagonal honeycomb structures for the first time [32], and derived Gibson’s formula:

(1) $$\begin{array}{c} E_{x}=E_{S}\frac{t^{3}}{l^{3}}\frac{\cos\theta }{(\beta +\sin\theta )\sin^{2}\theta }\\ \nu_{x}=\frac{\cos^{2}\theta }{(\beta +\sin\theta )\sin\theta }\\ E_{y}=E_{S}\frac{t^{3}}{l^{3}}\frac{\left(\beta +\sin\theta \right)}{\cos^{3}\theta }\\ \nu_{y}=\frac{(\beta +\sin\theta )\sin\theta }{\cos^{2}\theta }\\ G_{xy}=E_{S}\frac{t^{3}}{l^{3}}\frac{\left(\beta +\sin\theta \right)}{\beta^{2}(2\beta +1)\cos\theta } \end{array}\}$$

where $E_{S}$ is the elastic modulus of the honeycomb structure material; β=h/l according to Gibson’s formula, $\nu_{x}\nu_{y}=1$, so the elasticity matrix is uncertain, which makes it so Gibson’s formula cannot be directly applied. This is because the formula ignores the expansion deformation of the cellular structure wall in the process of derivation. According to Gibson’s idea of solving the mechanics of the honeycomb structure, the mechanical parameters corresponding to the stress state in the Y direction are derived. In the derivation process, the honeycomb structure is equivalent to a homogeneous structure.

**Figure 4:**
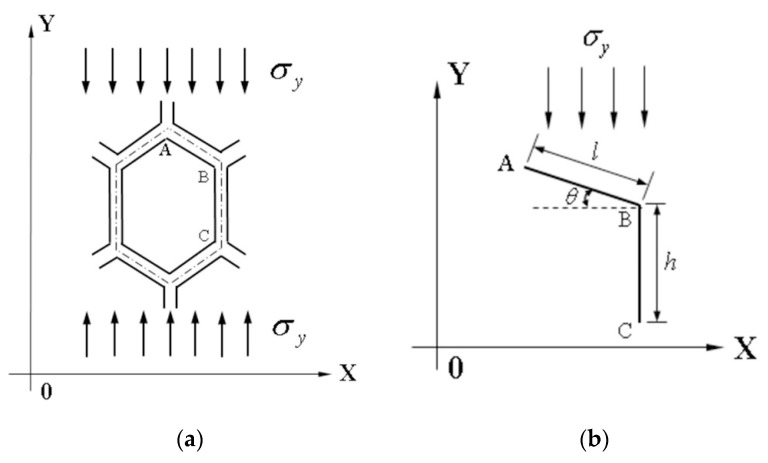

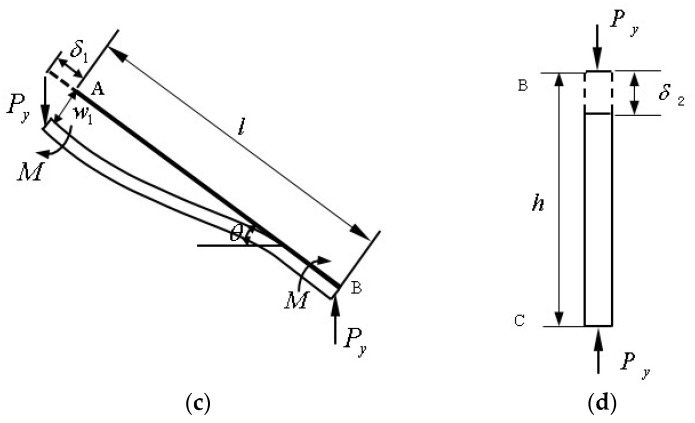
Schematics of tensile deformation of hexagonal honeycomb cell in Y direction: (**a**) compression of hexagonal honeycomb cell in the Y direction; (**b**) stress on ABC segment; (**c**) analysis of AB segment; and (**d**) analysis of BC segment.

When hexagonal honeycomb cells are in a unidirectional compression state in the Y direction, the homogeneous material model is assumed as shown in Figure 4a. Figure 4b shows the analysis of the ABC segment of hexagonal honeycomb cells, Figure 4c shows the deformation of the cellular wall—AB segment, and Figure 4d shows the vertical deformation of the cellular wall—BC segment [33,34].

According to the equilibrium condition of the force:

(2) $$M=\frac{1}{2}P_{y}l\cos\theta$$

where the force $P_{y}$ is

(3) $$P_{y}=\sigma_{cy}A_{y}=\sigma_{cy}l\cos\theta \cdot b$$

where:

M—the bending moment of the cellular node of the honeycomb, N⋅mm;
$P_{y}$—the external force of the cellular node of the honeycomb, N;
$A_{y}$—the cross-sectional area of the honeycomb cell under stress in the Y direction, ${\mathrm{mm}}^{2}$.

According to the beam bending theory of material mechanics, the deflection of cellular wall plate AB can be obtained as follows:

(4) $$w_{1}=\frac{P_{y}l^{3}\cos\theta }{12E_{s}I}$$

where I is the moment of inertia, $I=\frac{1}{12}bt^{3}$, which can be obtained by substituting into Equation (4):

(5) $$w_{1}=\frac{P_{y}l^{3}\cos\theta }{E_{s}bt^{3}}$$

According to Hooke’s law, under the action of external force $P_{y}$, the axial elongation of cellular wall plate AB can be expressed as

(6) $$\delta_{1}=\varepsilon_{AB}^{y}l=\frac{\sigma_{AB}^{y}}{E_{S}}l=\frac{P_{y}\sin\theta }{bt}\frac{l}{E_{s}}$$

where $\varepsilon_{AB}^{y}=\frac{\sigma_{AB}^{y}}{E_{S}}$ is the linear strain of cellular wall panel AB under the action of external force $P_{y}$. $\sigma_{AB}^{y}=\frac{P_{y}\sin\theta }{bt}$ is the normal stress of cellular wall panel AB on its cross-section.

The axial compression of cell cellular wall plate BC is:

(7) $$\delta_{2}=\varepsilon_{BC}^{y}h=\frac{\sigma_{BC}^{y}}{E_{S}}h=\frac{P_{y}}{bt}\frac{h}{E_{s}}$$

where $\varepsilon_{BC}^{y}=\frac{\sigma_{BC}^{y}}{E_{S}}$ is the linear strain of cellular wall panel BC under the action of external force $P_{y}$. $\sigma_{BC}^{y}=\frac{P_{y}}{bt}$ is the normal stress of cellular wall panel BC on its cross-section.

Therefore, the equivalent strain in the Y direction is

(8) $$\varepsilon_{cy}=\frac{\Delta l}{l}=\frac{w_{1}\cos\theta +\delta_{1}\sin\theta +\delta_{2}}{h+l\sin\theta }=\frac{P_{y}l^{3}(\cos^{2}\theta +\frac{t^{2}}{l^{2}}\sin^{2}\theta +\frac{t^{2}}{l^{3}}h)}{E_{S}bt^{3}(h+l\sin\theta )}$$

Let β=h/l; then, Formula (8) is

(9) $$\varepsilon_{cy}=\frac{P_{y}l^{2}\cos^{2}\theta [1+\frac{t^{2}}{l^{2}}(\tan^{2}\theta +\beta \sec^{2}\theta )]}{E_{S}bt^{3}(\beta +\sin\theta )}$$

According to the definition of Poisson’s ratio, the equivalent Poisson’s ratio $\nu_{cy}$ of honeycomb cells in the Y direction is

(10) $$\nu_{cy}=\left|\frac{\varepsilon_{cx}}{\varepsilon_{cy}}\right|=-\frac{\varepsilon_{cx}}{\varepsilon_{cy}}=\frac{(\beta +\sin\theta )\sin\theta }{\cos^{2}\theta }\times \frac{1-\frac{t^{2}}{l^{2}}}{1+(\tan^{2}\theta +\beta \sec^{2}\theta )\frac{t^{2}}{l^{2}}}$$

According to the definition of the elastic modulus, the equivalent elastic modulus $E_{cy}$ of honeycomb cells in the Y direction is

(11) $$E_{cy}=-\frac{\sigma_{cy}}{\varepsilon_{cy}}=E_{S}\frac{t^{3}}{l^{3}}\frac{\left(\beta +\sin\theta \right)}{\cos^{3}\theta }\times \frac{1}{1+(\tan^{2}\theta +\beta \sec^{2}\theta )\frac{t^{2}}{l^{2}}}$$

When the θ value is equal to 0° and −30°, the square cell and honeycomb cell can be obtained, and the corresponding $E_{cy}$ can be obtained by substituting β and t as studied in this paper. The results obtained are consistent with the research of Li Xiang [35].

### 4.2. Structural Compressive Behavior

Compressive strength refers to the ability of structural materials to resist deformation and failure when subjected to compressive force. It is an important index by which to measure the compression performance of structural materials, and is usually used to evaluate the stability and reliability of structural materials under pressure. The specific formula used to calculate the compressive strength can be defined as:

(12) $$\sigma =\frac{F_{\max}}{l\cdot w}$$

where *F*_max_ is the maximum compression load, *l·w* is the cross-sectional area of the load application, *l* is the length of the specimens, and *w* is the width of the specimens.

The specific strength is mainly a performance index that indicates the strength and bearing capacity of structural materials per unit mass or unit volume.

The specific strength is the strength of a structure material divided by its apparent density. The specific strength can be defined as:

(13) $$\sigma_{ss}=\frac{\sigma }{\rho *}$$

where ρ* is the apparent density of the specimen structure. The apparent density is the ratio of the mass of a specimen to its apparent volume. When calculating it in this article, the apparent volume is directly measured by measuring the volume of the specimen.

The load–mass ratio (LMR) is a performance index that describes the mass distribution proportion of structural materials under a specific load. While meeting the structural strength and stiffness, the weight of the structure should be reduced as much as possible. This is of great significance in improving the overall performance of the structure and reducing energy consumption. The specific weight-to-resistance ratio can be defined as:

(14) $$\mathrm{LMR}=\frac{F_{\max}}{m}$$

where LMR is the load–mass ratio, and *m* is the mass of the specimens.

Energy absorption refers to the ability of structural materials to absorb and dissipate energy when subjected to external forces. The energy absorbed can be defined as:

(15) $$EA=\int_{0}^{\varepsilon_{d}}\sigma \times \varepsilon d\varepsilon$$

where σ and ε are the nominal stress and strain, respectively, and $\varepsilon_{d}$ the densification strain.

### 4.3. Finite Element Analysis

The finite element analysis is based on Auto Inventor to predict the deformation process of four types of thickness gradient honeycomb structures under quasi-static compression. The geometric parameters of the finite element analysis model are the same as those of the experimental specimens. The experimental material properties shown in Table 4 were defined in the finite element simulation.

During the simulation process, the fixed plates of the structure are made of structural steel, and the specimens are placed between the upper and lower fixed plates. The bottom surface of the structural model is fixed to the lower plate of the fixed plate, and the load is subjected to the upper plate of the fixed plate. The model is connected with the upper and lower fixing plates by separating but without sliding. With the quasi-static compression simulation, it is difficult to quantitatively describe the elastic–plastic changes of structural specimens. Therefore, this paper can only qualitatively analyze the stress change state of the specimens under a flat compression load. The entity unit selected for the model is C3D4. Based on the load-bearing capacity of four types of honeycomb structure specimens, we analyze the stress distribution of the four types of structural specimens.

**Figure 5:**
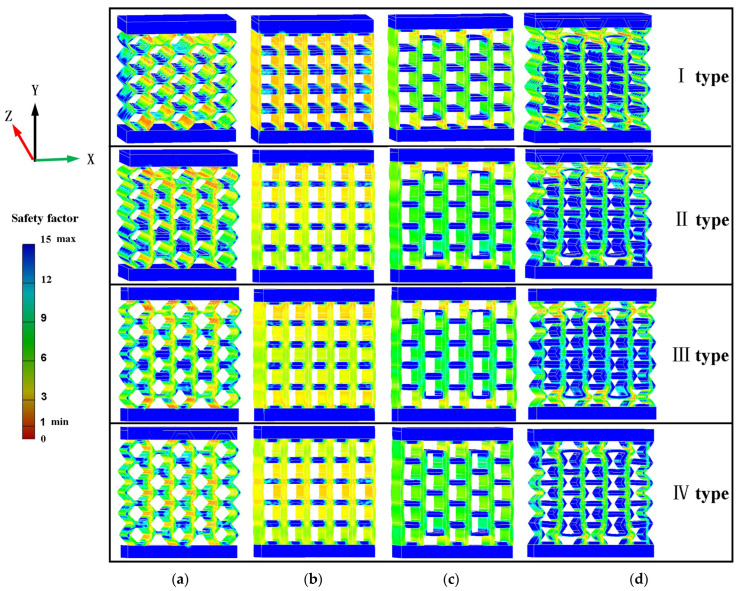
Safety factor distribution of the simulation structure: (**a**) hexagonal honeycomb; (**b**) square honeycomb; (**c**) quasi-square honeycomb; and (**d**) re-entrant hexagonal honeycomb.

In the structural design, the safety factor is usually used to reflect the safety degree of the structure. Therefore, the structural failure state and failure order can be judged from the safety factor. The safety factor distribution of the simulated specimens with four structural types is shown in Figure 5. In the simulation results, the failure of the honeycomb structure model mainly occurs on the inner wall of the cell. The maximum danger of the type I structure occurs at the upper and lower ends of the model. The failure of type II structures mainly occurs in the upper part of the model, because the cellular wall thickness of the type II structure is gradually increasing. The failure of the type III structure mainly occurs at the upper and lower ends of the model. The type IV structure has stress distribution in each cell, but the danger factor is relatively small compared to the previous three structures. The deformation of cell walls in both square honeycomb structures and quasi-square honeycomb structures is significant. The failure of square honeycomb structures mainly occurs on the cell’s side walls, and the horizontal cell walls bear relatively small stresses. The failure of quasi-square honeycomb structures mainly occurs at the upper and lower ends of the model, and the vertical cell walls are under stress. The failure of re-entrant honeycomb structures mainly occurs at the upper and lower ends of the structure.

The simulation results show that, under the same stress, the type I structure has the highest risk factor and the type IV structure has the lowest risk factor. The configuration of the structure, the wall thickness of the cells in the structure, and the arrangement order of the cells have a significant impact on the load-bearing capacity of the structure. The thinnest part of the cellular wall in the structure shows the highest risk factor. When the cellular wall thickness is the same in the structure, the danger coefficient is highest at the upper and lower ends of the structure.

## 5. Results and Discussion

### 5.1. Structural Failure Process

**Figure 6:**
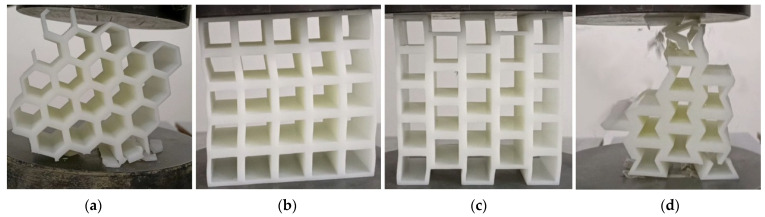
Tensile failure and deformation modes of specimens: (**a**) hexagonal honeycomb; (**b**) square honeycomb; (**c**) quasi-square honeycomb; and (**d**) re-entrant hexagonal honeycomb.

The failure and deformation of the four honeycomb structure specimens are shown in Figure 6. In Figure 6, the failure of the specimens in both the (a) and (d) structures starts from the upper and lower cellular walls of the specimens, and the main form of cellular wall failure is strip fragments. The specimens of structures (b) and (c) have a large deformation and show great bending flexibility when subjected to flat compression load. The cellular walls on the left and right sides of the specimens are thin and have a large bending deformation, while the cell deformation in the middle part is relatively small.

### 5.2. Gradient Honeycomb Structure

**Figure 7:**
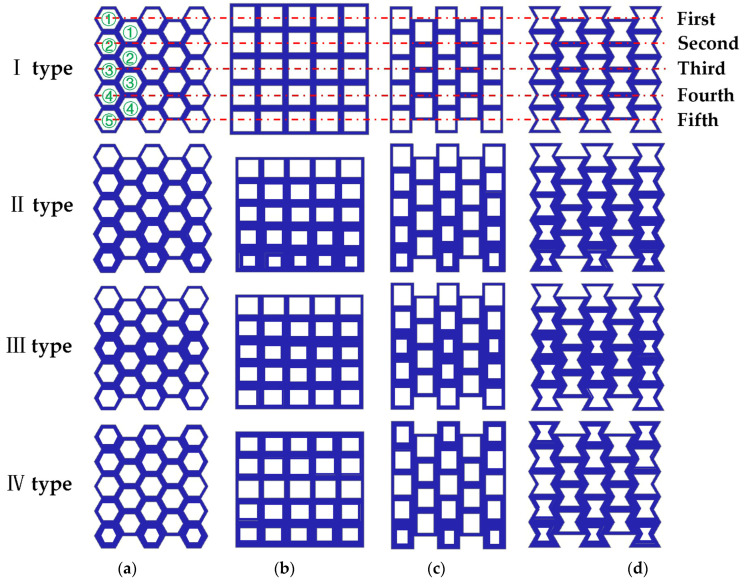
Four types of thickness gradient honeycomb structures: (**a**) hexagonal honeycomb; (**b**) square honeycomb; (**c**) quasi-square honeycomb; and (**d**) re-entrant hexagonal honeycomb.

Up to now, most honeycomb structural materials are formed by the repeated arrangement of cells with the same geometric parameters. The mechanical properties of honeycomb structural materials mainly depend on the structural type of the structural cells and the geometric parameters of the structural configuration. Therefore, cells with the same geometric parameters usually produce uniform structural properties [36]. However, in some cases, structures with gradient properties have extraordinary advantages over those with uniform properties. For example, in nature, structures or materials with gradient mechanical properties exist widely, such as bamboo, wood, and bone. The number of cells or pores of these structures or materials are distributed in a specific hierarchical gradient to adapt to complex environments. Gradient structures make them flexible and strong [37]. Artificial gradient structures or materials have also been widely used, mainly by changing the density or thickness of materials [38]. Zhixi Guo et al. studied the multi-arc negative-Poisson’s-ratio structure cell. The experimental study showed that the thickness change in the cellular wall had a significant impact on the energy absorption capacity of the thickness gradient honeycomb structure [39]. In the design of car seats, the thickness of the local area of the seat is increased according to the maximum strain position and the stress distribution law of the seat, the reinforced rib plate is established, and the material thickness is appropriately reduced at the position with a small deformation. The weight of the optimized seat plastic panel is reduced by 15% [40]. Compared with the design of changing density, the gradient structure design of changing thickness is easier to realize. Therefore, the gradient structure design of hexagonal, square, and re-entrant honeycomb structures is carried out in this paper in order to study their mechanical properties. Four types of thickness gradient honeycomb structures are shown in Figure 7. The four types of honeycomb structures shown in Figure 7 have a total of five layers of honeycomb cells from top to bottom. The top layer is the first layer, arranged from top to bottom, and the bottom layer is the fifth layer. The middle part of the other three structures, except for the square honeycomb, has a total of four layers, arranged from top to bottom. The top layer is the first layer, and the bottom layer is the fourth layer. In Figure 7, green ① is used to represent the first layer, and red dots and lines are used to indicate the same number of layers for the four honeycomb structures.

The printing process of the 3D printer used in this experiment is a layer-by-layer growth mode, and the thickness of each layer is 0.1 mm. The length, width, and height dimensions of each type of specimen shown in Figure 6 are the same as those in Table 2, except for difference in wall thickness. The cell wall thickness of the specimens increased by 0.2 mm. The wall thickness dimensions of four types of thickness gradient honeycomb structure specimens are shown in Table 5. The designed gradient thickness honeycomb structure is made of photosensitive resin (DSM8000). According to the ASTM C365-16 standard, uniaxial quasi-static compression tests were carried out on the specimens using a general mechanical testing machine (Model WDW-50, Changchun Kexin Testing Instrument Co., Ltd., Changchun, China) at room temperature at a displacement rate of 1 mm/min.

### 5.3. Uniaxial Compression Results

**Figure 8:**
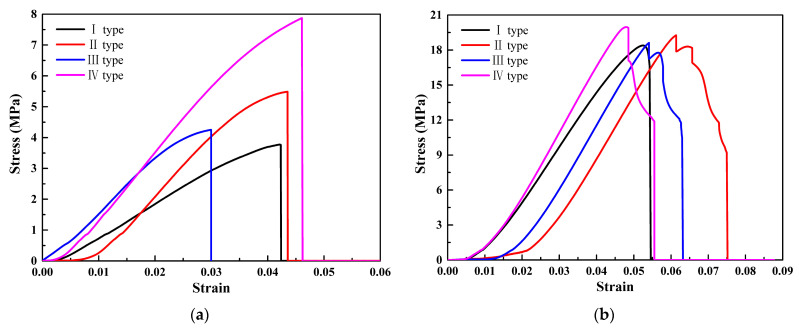

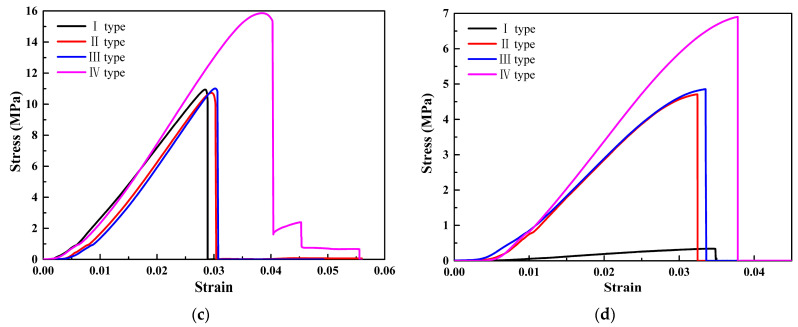
Stress–strain curves of four gradient honeycomb structures: (**a**) hexagonal honeycomb; (**b**) square honeycomb; (**c**) quasi-square honeycomb; and (**d**) re-entrant hexagonal honeycomb.

The stress–strain curves of four types of thickness gradient honeycomb structure specimens are shown in Figure 8. From Figure 8, it can be observed that the stress and strain of the square honeycomb structure specimen are the highest, while the stress and strain of the re-entrant hexagonal honeycomb are the lowest. The load-bearing capacity and structural deformation of the square honeycomb structure specimen are the highest. The slope of the stress–strain curve represents the elastic modulus of the specimens during the elastic stage. Therefore, it can be concluded that the elastic modulus of the square honeycomb structure specimen is the highest, while the elastic modulus of the re-entrant hexagonal honeycomb is the lowest. The area under the stress–strain curve is usually used to evaluate the fracture toughness of the specimens. Therefore, it is concluded that the fracture toughness of the quadrate honeycomb structure type II specimens is the largest. Due to the existence of the thickness gradient structure, the specimens will show uneven deformation behavior under load. The deformation of structural non-uniformity manifests as different slopes or shapes on the stress–strain curve. Therefore, it is concluded that the shape of the quasi-square honeycomb structure type IV specimens changes greatly compared with the other three structural types.

Through a comparative analysis of the stress–strain curves of four thickness gradient honeycomb structure specimens, it was found that the structure with the same cell wall thickness among the four honeycomb structures had the lowest load-bearing capacity. The increase in the cell wall thickness improved the load-bearing capacity of the honeycomb structures to varying degrees. The increase in the cell wall thickness at the top and bottom ends of the honeycomb structures can effectively enhance the load-bearing capacity of the structures. The load-bearing capacities of the square honeycomb structure and the quasi-square honeycomb structure are both higher than the other two types of structural specimens. This is because the cells of the square honeycomb structure and the quasi-square honeycomb structure are square, and the square structure is a structure with a zero Poisson’s ratio. When it is subjected to external force, the ratio of transverse strain to longitudinal strain is zero; that is, the transverse size of the structure remains basically unchanged during tension or compression. It is precisely because of this characteristic of the cell that the square honeycomb structure and the quasi-square honeycomb structure can effectively disperse the stress, reduce the stress concentration phenomenon, and improve the bearing capacity of the material.

### 5.4. Compression Performance

We conduct flat compression tests on specimens with four types of structures, and compare and analyze their mechanical properties. H represents the honeycomb structure, S represents the square honeycomb structure, Q represents the quasi-square honeycomb structure, and R represents the re-entrant honeycomb structure. The error bars represent the standard error of the mean. The error bars for the four structures are represented in four colors, with black representing the honeycomb structures, red representing the square honeycomb structures, blue representing the quasi square structures, and pink representing the re-entrant honeycomb structure. I type structures are represented by lines at a 45 ° angle to the horizontal direction, II type structures are represented by grid lines, III type structures are represented by horizontal lines, and IV type structures are represented by vertical lines.

The results of the flatwise compressive tests are listed in Table 6. The compressive strength and specific strength of square honeycomb structures are significantly higher than those of the other three types of honeycomb structures.

**Figure 9:**
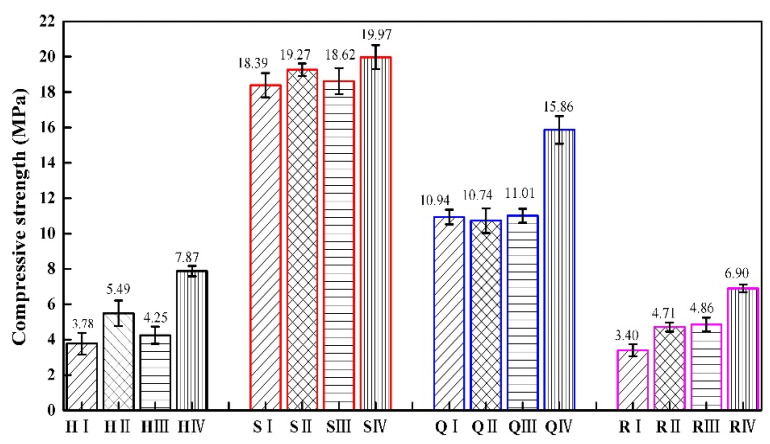
The compressive strength of four gradient honeycomb structures.

Figure 9 shows the compressive strength of different types of specimens of these four structures. The compressive strength of the square honeycomb structure is greater than that of the quasi-square honeycomb structure. Zaharia et al. [41] rotated the square honeycomb structure by 45° and referred to it as a diamond structure. They conducted compressive strength tests on it, and the results were consistent with those in this paper. The research results of Nath et al. [42] also indicate that diamond structures have a greater compressive strength. The compressive strength of the honeycomb structure is greater than that of the re-entrant hexagonal honeycomb structure. The research results of Zhao Junhong et al. [8] are consistent with our findings.

The compressive strength of the square honeycomb structure is the highest, followed by that of the quasi-square honeycomb structure. This is because the overall deformation of the square honeycomb structure is large in the process of plane compression, which can effectively disperse the pressure. While the deformation of the quasi-square honeycomb structure is relatively small, and the pressure dispersion of the whole structure is small, which reduces the compression strength of the quasi-square honeycomb structure. The compressive strength of the re-entrant honeycomb structure is affected by its apparent area. When the average bearing capacity of the re-entrant honeycomb structure is higher than that of the honeycomb structure, the apparent area of the re-entrant honeycomb structure is larger than that of the honeycomb structure, resulting in the higher compressive strength of the honeycomb structure.

Compared to similar structures, the compression strength of the H IV type is 108.20% higher than that of the H I type, the compression strength of the S IV type is 8.59% higher than that of the S I type, the compression strength of the Q IV type is 44.97% higher than that of the Q I type, and the compression strength of the R IV type is 102.94% higher than that of R I type. In the same type of structure, the thickness of the cellular wall in the structure has a positive impact on the compressive strength of the structure. The greater the thickness of the cellular wall, the greater the overall stiffness of the structure. Therefore, the specimens with the same cellular wall thickness has the lowest compressive strength among the four structures.

**Figure 10:**
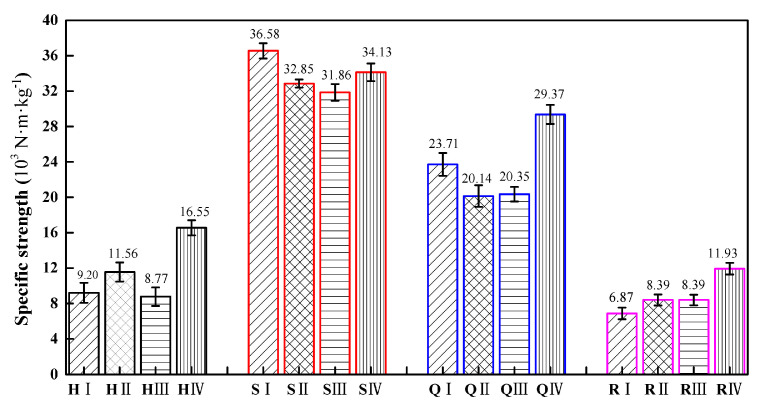
The specific strength of four gradient honeycomb structures.

Figure 10 shows the specific strength of different types of specimens of the four structures. It can be observed from Figure 10 that the specific strength of the square honeycomb structure is the largest, followed by the quasi-square honeycomb structure, and the re-entrant honeycomb structure is the smallest. This result is consistent with the research findings of Nath et al. [42]. A high specific strength means that, under the same volume, this type of structure can withstand a greater load, and has better bearing capacity and anti-failure performance. Compared to similar structures, the specific strength of the H IV type is 79.89% higher than that of the H I type, the specific strength of the Q IV type is 23.87% higher than that of the Q I type, and the specific strength of the R IV type is 73.65% higher than that of the R I type.

According to the simulation diagram of the safety factor distribution in Figure 5, the specific strength of the structure is directly related to the arrangement of cells in the structure and the distribution and transfer path of stress in the structure. Only the stress of the SⅠ structure is uniformly distributed on the side wall of the cell, so the specific strength of this structure type is the highest. In the square and quasi-square graded thickness honeycomb structures, the orderly arrangement of square cells can make the stress relatively more evenly distributed on the constituent cells of the structure, which can improve the overall specific strength of the structure.

The experimental results of the load−mass ratio and energy absorbed are shown in Table 7. The load-to-mass ratio and energy absorption of the square honeycomb structure are the highest among the four types of structures.

**Figure 11:**
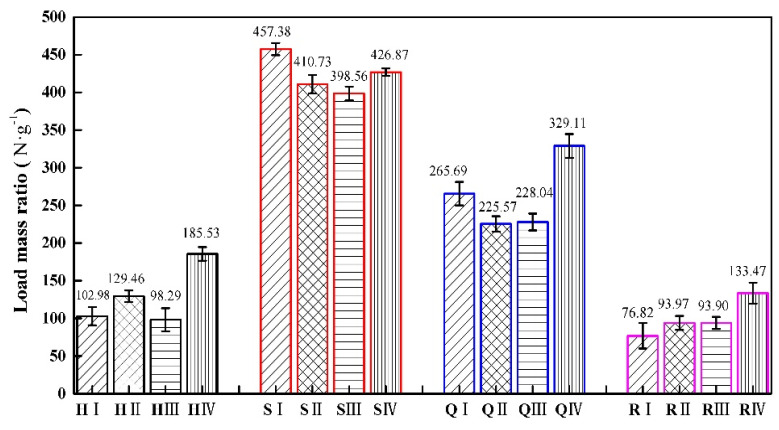
The load−mass ratio of four gradient honeycomb structures.

Figure 11 shows the load−mass ratio of different types of specimens in four structures. The load−mass ratio reflects the balance between the mass and performance of the structure under load. The lighter structure has a higher load−mass ratio under the same load. In this regard, the square and quasi-square thickness gradient honeycomb structures with square cells have obvious advantages, because they can achieve a lighter weight while ensuring a certain performance. It can be observed from Figure 11 that the load−mass ratio of the square honeycomb structure is the largest, followed by the quasi-square honeycomb structure, and the re-entrant honeycomb structure is the smallest. In the square thickness gradient honeycomb structure, the load–mass ratio of the cell arrangement structure with an equal wall thickness is the highest. Compared to similar structures, the load−mass ratio of the H IV type is 80.16% higher than that of the H I type, the load−mass ratio of the Q IV type is 23.87% higher than that of the Q I type, and the load−mass ratio of the R IV type is 73.47% higher than that of the R I type.

In the thickness gradient honeycomb structure with the same shape, changing the arrangement order of the cells will have a significant impact on the overall load−mass ratio of the structure. A reasonable arrangement can make the mass more evenly distributed on the structure, so as to reduce the center of gravity and reduce the overturning moment under load. Therefore, by effectively changing the cell order in the same type of structure, the thickness gradient honeycomb structure can optimize the mass distribution of the structure, which is helpful in improving the load−mass ratio of the structure.

**Figure 12:**
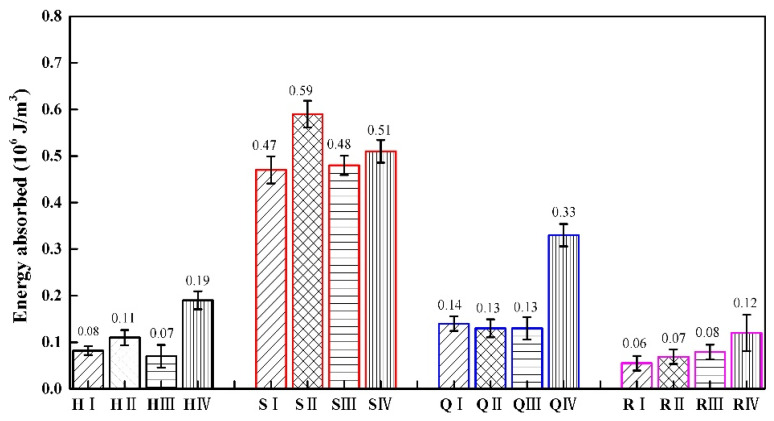
The energy absorbed of four gradient honeycomb structures.

Figure 12 shows the energy absorption of different types of specimens in the four structures. It can be observed from Figure 12 that the square honeycomb structure has the largest energy absorption, followed by the quasi-square honeycomb structure, and the re-entrant honeycomb structure has the smallest energy absorption. In a square thickness gradient honeycomb structure, the S II type structure has the highest energy absorption. Among the four thickness gradient honeycomb structures, the type IV structure has good energy absorption. This result is consistent with the research findings of Shu Cai Xu et al. [43]. Compared to similar structures, the energy absorption of the H IV type is 137.5% higher than that of the H I type, the energy absorption of the S IV type is 8.51% higher than that of the S I type, the energy absorption of the Q IV type is 135.71% higher than that of the Q I type, and the energy absorption of the R IV type is 100%higher than that of the R I type.

It can be concluded that the energy absorption capacity of the structural materials is closely related to their structural design and the mechanical properties of the cellular materials. The thickness of the cellular wall and the order of the cell arrangement will affect the energy absorption and dispersion ability of the structure under load. Different cell arrangements and wall thicknesses can be designed according to different application scenarios in engineering, which can more effectively disperse energy into multiple cells, reduce local damage, and improve the stability of the overall structure.

## 6. Conclusions

Four types of honeycomb structure specimens with a thickness gradient were designed and manufactured by 3D-printing technology. The fabricated specimens have been subjected to quasi-static compression tests. Based on the current theoretical analysis, finite element simulation, and experimental research, the following conclusions are drawn:

(1) Among the four kinds of thickness gradient honeycomb structures with periodic regular structures, the thickness gradient honeycomb structure with square cells has the best overall mechanical properties and the highest bending flexibility under pressure. By changing the thickness of the cellular wall and the arrangement order of the cells, the corresponding mechanical properties will also change. Increasing the thickness of the cellular wall at the upper and lower ends of the thickness gradient honeycomb structure can effectively improve the bearing capacity of the structure.
(2) The thickness of the cellular wall in the honeycomb structure directly affects the overall stiffness of the gradient honeycomb structure. The thicker the cellular wall, the stronger the deformation resistance of the honeycomb structure under external force, that is, the greater the stiffness of the structure. Therefore, increasing the thickness of the cellular wall can improve the overall stiffness of the honeycomb structure, so as to improve its mechanical strength.
(3) The order of the cell arrangement will directly affect the mass distribution, center of gravity position, stress distribution, and transmission path of the honeycomb structure. Optimizing the arrangement of the cells can reduce the position of the center of gravity of the structure, reduce the overturning moment under the load state, and make the stress more evenly distributed among the cells, which can effectively improve the bearing capacity of the honeycomb structure and cause it to have a higher structural stability.
(4) The mechanical properties of structures with different Poisson’s ratios are significantly different. The hexagonal thickness gradient honeycomb structure is a positive-Poisson’s-ratio structural material, and its compressive strength is related to the structural configuration and material density; the square gradient honeycomb structure specimen and the quasi-square gradient honeycomb structure specimen are zero-Poisson’s-ratio structural materials, which have a high compressive strength, and the cell configuration of the structure can effectively disperse and resist external pressure; however, the re-entrant thickness gradient honeycomb structure specimen is a negative-Poisson’s-ratio structural material, and its compressive strength is low in the flat compression state. At the same time, its bearing capacity and compressive strength are more easily affected by the internal cell configuration of the structure.

The 3D-printed thickness gradient honeycomb structure is a new type of lightweight and high-strength structure, which can be used for the façade walls, sloping roofs, ceilings, etc. of landscape houses in building structures. In daily life, it can be used for household kitchen cabinet panels, furniture shelves, and other daily necessities. Placing insulation, noise reduction, sound absorption, and other materials in the gaps of cellular structures can help maintain a stable indoor temperature, improve indoor comfort, and reduce building energy consumption. It will achieve the integration of structure and function, and it will have broad application prospects. In the future, multi-material printing can be achieved in order to manufacture more complex gradient honeycomb structures, and intelligent design can be adopted to design more efficient and stable structures through computer simulation and optimization.

## Figures and Tables

**Table 1:** Geometric parameters of three basic models with the same porosity.

Unit Cell	Side Length L_1_/mm	Side Length L_2_/mm	Included Angle θ/(°)
Honeycomb	4.00	4.00	30.00
Square honeycomb	6.00	6.93	90.00
Re-entrant honeycomb	8.00	4.00	30.00

**Table 2:** Size of the specimens.

Name	Height (h) mm	Width (w) mm	Length (*l*) mm
Honeycomb	89.28	82.48	50.00
Square honeycomb	80.00	89.30	50.00
Quasi-square honeycomb	89.30	80.00	50.00
Re-entrant honeycomb	89.28	94.02	50.00

**Table 3:** Printing parameters of 3D printer iSLA660.

Serial Number	Parameter	Value
1	Laser wavelength	354.7 nm
2	Recoating thickness	0.1 mm
3	Spot diameter	0.15 mm
4	Part scanning speed	6.0 m/s
5	Jumping speed of parts	10.0 m/s
6	Repetitive positioning accuracy	±0.01 mm
7	Temperature	20 °C
8	Volume of resin vat	200 kg

**Table 4:** Material properties of the photosensitive resin DSM8000 (Unit: MPa).

Compressive Modulus	Compressive Strength	Flexural Modulus	Flexural Strength	Tensile Modulus	Tensile Strength
1699.97 ± 169.08	53.17 ± 7.28	1897.30 ± 134.24	59.29 ± 7.11	1205.02 ± 213.02	36.46 ± 2.66

**Table 5:** Wall thickness of four types of thickness gradient honeycomb structure (unit: mm).

Wall Thickness		Honeycomb Type		
	I	II	III	IV
First	2.00	2.00	2.00	2.60
Second	2.00	2.20	2.40	2.20
Third	2.00	2.40	2.80	2.00
Fourth	2.00	2.60	2.60	2.40
Fifth	2.00	2.80	2.20	2.80

**Table 6:** Experimental results of compressive strength and specific strength.

Type	Compressive Strength (MPa)		Specific Strength (MPa)	
	Mean Value ± Standard Deviation	Coefficient of Variation (%)	Mean Value ± Standard Deviation	Coefficient of Variation (%)
H I	3.78 ± 0.61	16.14	9.20 ± 1.12	12.17
H II	5.49 ± 0.72	13.11	11.56 ± 1.09	9.43
H III	4.25 ± 0.48	11.29	8.77 ± 1.04	11.86
H IV	7.87 ± 0.29	3.68	16.55 ± 0.87	5.26
S I	18.39 ± 0.69	3.75	36.58 ± 0.87	2.38
S II	19.27 ± 0.35	1.82	32.85 ± 0.46	1.40
S III	18.62 ± 0.68	3.65	31.86 ± 0.94	2.95
S IV	19.97 ± 0.74	3.71	34.13 ± 1.01	2.96
Q I	10.94 ± 0.40	3.66	23.71 ± 1.29	5.44
Q II	10.74 ± 0.70	6.52	20.14 ± 1.23	6.11
Q III	11.01 ± 0.39	3.54	20.35 ± 0.82	4.03
Q IV	15.86 ± 0.78	4.92	29.37 ± 1.09	3.71
R I	3.40 ± 0.34	10.00	6.87 ± 0.65	9.46
R II	4.71 ± 0.26	5.52	8.39 ± 0.63	7.51
R III	4.86 ± 0.78	16.05	8.39 ± 0.59	7.15
R IV	6.90 ± 0.22	3.19	11.93 ± 0.67	5.62

Note: The numbers in the table represent the mean ± standard deviation; The application of one-way ANOVA showed significant differences (*p* < 0.001).

**Table 7:** Experimental results of the load−mass ratio and energy absorbed.

Type	Load–Mass Ratio (N·g^−1^)		Energy Absorbed (10^6^ J/m^3^)	
	Mean Value ± Standard Deviation	Coefficient of Variation (%)	Mean Value ± Standard Deviation	Coefficient of Variation (%)
H I	102.98 ± 12.01	11.66	0.08 ± 0.0093	11.63
H II	129.46 ± 7.73	5.97	0.11 ± 0.0161	14.64
H III	98.29 ± 15.22	15.48	0.07 ± 0.0241	34.40
H IV	185.53 ± 9.02	4.86	0.19 ± 0.0192	10.13
S I	457.38 ± 8.04	1.76	0.47 ± 0.0291	6.19
S II	410.73 ± 12.18	2.97	0.59 ± 0.0286	4.85
S III	398.56 ± 9.18	2.30	0.48 ± 0.0207	4.31
S IV	426.87 ± 4.89	1.15	0.51 ± 0.0241	4.73
Q I	265.69 ± 15.61	5.88	0.14 ± 0.0158	11.29
Q II	225.57 ± 10.05	4.46	0.13 ± 0.0192	14.77
Q III	228.04 ± 11.19	4.91	0.13 ± 0.0241	18.54
Q IV	329.11 ± 15.71	4.77	0.33 ± 0.0241	7.27
R I	76.82 ± 16.88	21.97	0.06 ± 0.0158	26.33
R II	93.97 ± 9.13	9.72	0.07 ± 0.0156	22.29
R III	93.90 ± 8.12	8.65	0.08 ± 0.0155	19.38
R IV	133.47 ± 13.77	10.32	0.12 ± 0.0389	32.42

Note: The numbers in the table represent the mean ± standard deviation; The application of one-way ANOVA showed significant differences (*p* < 0.001).

## Data Availability

The study did not report any data that are available upon reasonable request.
